# The potential roles of gut microbiome in porto-sinusoidal vascular disease: an under-researched crossroad

**DOI:** 10.3389/fmicb.2025.1556667

**Published:** 2025-03-03

**Authors:** Yangjie Li, Lingna Lyu, Huiguo Ding

**Affiliations:** Department of Gastroenterology and Hepatology, Beijing Youan Hospital Affiliated with Capital Medical University, Beijing, China

**Keywords:** porto-sinusoidal vascular disease, portal hypertension, gut-liver axis, gut microbiome, microvascular thrombosis

## Abstract

Accumulating evidence indicates that patients with liver diseases exhibit distinct microbiological profiles, which can be attributed to the bidirectional relationship of the gut-liver axis. Porto-sinusoidal vascular disease (PSVD) has recently been introduced to describe a group of vascular diseases of the liver, involving the portal venules and sinusoids. Although the pathophysiology of PSVD is not yet fully understood, several predisposing conditions, including immunodeficiency, inflammatory bowel disease, abdominal bacterial infections are associated with the increasing in intestinal permeability and microbial translocation, supporting the role of altered gut microbiota and gut-derived endotoxins in PSVD etiopathogenesis. Recent studies have proposed that the gut microbiome may play a crucial role in the pathophysiology of intrahepatic vascular lesions, potentially influencing the onset and progression of PSVD in this context. This review aims to summarize the current understanding of the gut microbiome's potential role in the pathogenesis of hepatic microvascular abnormalities and thrombosis, and to briefly describe their interactions with PSVD. The insights into gut microbiota and their potential influence on the onset and progression of PSVD may pave the way for new diagnostic, prognostic, and therapeutic strategies.

## 1 Introduction

Porto-sinusoidal vascular disease (PSVD) is a rare vascular and parenchymal liver disease encompassing a spectrum of often subtle hepatic microvascular lesions and related microarchitectural abnormalities in the absence of cirrhosis in liver biopsy, regardless of the presence of portal hypertension (Premkumar and Anand, 2024). Previously, it was referred to as idiopathic non-cirrhotic portal hypertension (INCPH), characterized by the presence of portal hypertension in the absence of a clear underlying liver disease and portal vein thrombosis (PVT) (Lee et al., 2016). However, these diagnostic criteria for INCPH have certain shortcomings. INCPH excludes patients in the early stages of the disease spectrum who have not yet achieved portal hypertension but already exhibit histopathological lesions in sinusoids and portal venules (Khanna and Sarin, 2014). Similarly, nearly 40% of INCPH patients experience PVT as the disease progresses (Siramolpiwat et al., 2014). Excluding patients with PVT fails to acknowledge that PVT may be both a consequence and a contributing factor in the progression of INCPH. To address these limitations and facilitate early diagnosis, the Vascular Liver Disease Group recently introduced a novel entity named PSVD (De Gottardi et al., 2019). PSVD includes patients who meet the diagnostic criteria for INCPH but do not exhibit symptoms of portal hypertension as well as those with PVT or other causes of liver disease if the liver biopsy suggests PSVD, indicating that its prevalence may be significantly higher than that of INCPH. However, patients with PSVD are generally asymptomatic unless they present a complication typical of portal hypertension (Aggarwal et al., 2013; Kang et al., 2021). The diagnosis of PSVD remains challenging, which primarily relies on clinical signs of portal hypertension combined with specific histological features involving the porto-sinusoidal vascular abnormalities (obliterative portal venopathy, portal tract hypervascularization, and abnormal periportal vessels) as well as parenchymal abnormalities (Gioia et al., 2024; De Gottardi et al., 2022). Moreover, the pathophysiology responsible for PSVD is complex and hinders the development of treatments capable of altering the natural history of the disease. A better insight into the biological processes and pathophysiological mechanisms involved in PSVD is essential for identifying disease drivers and developing new diagnostic and therapeutic strategies.

Gut microbiome plays critical roles in the development of several vascular disease phenotypes by activating vascular endothelial cells, platelets, and innate immune cells (Hasan et al., 2020). Since the liver yields most of its blood supply via the portal circulation, the hepatic microcirculation constantly encounters gut-derived components, metabolites, and signals. These factors can induce changes in the liver sinusoidal endothelium, affecting the immune partitioning of the sinusoids and influencing portal hypertension (Kiouptsi et al., 2023). Although the exact pathogenesis of PSVD remains unclear, it is hypothesized to result from injuries and occlusion of the intrahepatic portal microvasculature, leading to increased resistance to portal blood flow and subsequent presinusoidal type of portal hypertension (Jin and Choi, 2023). Predisposing conditions of PSVD are related to immune disorders, infections, prothrombotic conditions, congenital or hereditary defects, drug exposure, and inherited vascular remodeling disorders (Kmeid et al., 2021). The link between gut microbiome, portal hypertension, and predisposing conditions of PSVD have supported that gut microbiota translocation into the sinusoids may impact on the pathophysiology of PSVD (Fiordaliso et al., 2023). Research has shown that intestinal permeability and gut-derived endotoxins play an important role in the pathogenesis of PSVD by activating the immune response of the liver, trigger inflammatory reactions in the liver, and thereby affect the health of the portal vein and sinusoidal vessels (Baffy and Portincasa, 2024). Previous studies reported that intestinal relocation with *Escherichia coli* (*E. coli*) might cause recurrent septic embolization leading to endothelial damage and the obstruction of small portal veins contributing to idiopathic portal hypertension (Kono et al., 1988; Giuli et al., 2023; Sarin and Aggarwal, 1998).

Recent advances in metagenomics and bioinformatics have provided new insights into the microbial ecology in different liver diseases (Nychas et al., 2025; Parthasarathy et al., 2024; Oh et al., 2020). Emerging studies have revealed the connection of intestinal microbiome and porto-sinusoidal vascular abnormalities, as well as hepatic thrombosis. In this review, we provide an overview of current knowledge regarding the role of the gut microbiome in the pathogenesis of intrahepatic microvascular abnormalities and thrombosis formation. Additionally, this review also introduces a brief description of the state of research and perspectives on the interactions between gut microbiome and PSVD progression. The insights into gut microbiota and its potential role in PSVD will help to elucidate the mechanism by which the gut microbiota influence PSVD and provide new opportunities for its diagnosis, prognosis, and treatment.

## 2 The potential interlink between gut microbiome and PSVD

The human gastrointestinal tract harbors over trillions of microorganisms including bacteria, fungi, viruses, and archaea that make up the gut microbiome (Hsu and Schnabl, 2023). The gut and liver have a symbiotic relationship with gut microbiome, which is referred to as the gut-liver axis (Wang et al., 2021b). The composition and structure of gut microbiota, intestinal barrier, liver vascular system, and liver status all play crucial roles in maintaining homeostasis within this axis. Under normal physiological conditions, the intestinal barrier in the gut liver axis, including physical (gut vascular and epithelial cell tight junctions), immunological (gut-associated lymphoid tissue), and biochemical (antimicrobial peptides, secretory immunoglobulin A and mucus layer) components (Tranah et al., 2021), forms the first line of defense for human immune system, while the liver vascular microenvironment provides a second line of defense to preventing the pathogenic factors of intestinal mucosal immune response triggering the dissemination of systemic inflammatory (He et al., 2021; Seo and Shah, 2012). A perturbation of this balance causes gut dysbiosis, which not only leads to liver damage and systemic inflammation but is also related to impaired microcirculation, abnormal vascular permeability, and liver hemodynamics (Simbrunner et al., 2019).

PSVD involves abnormalities in the liver's vascular system and is likely a group of different diseases that can cause inflammation and obstruction of porto-sinusoidal vascular system affect portal venous pressure (Isidro and Zhao, 2023). Most of PSVD patients appears idiopathic portal hypertension, and relevant pathogenesis is involved in liver structural distortion fibrosis, microvascular thrombosis, dysfunction of cellular elements in the hepatic sinusoidal vascular microenvironment (Mehta et al., 2014). The portal vein is frequently exposed to intestinal microbe-associated pathogen-associated molecular patterns (PAMPs), including lipopolysaccharide (LPS), antigens, as well as bacteria, and transmits them to the liver, thus eliciting negative effects on the liver (Pabst et al., 2023). Gut dysbiosis can disrupt intestinal barriers, increasing permeability causing the translocation of PAMPs into the liver through portal vein and participate in enterohepatic circulation (Spadoni et al., 2015), which results in an imbalance of the gut-liver-vascular homoeostasis, activation pro-inflammatory response in hepatic sinus and increasing hepatic vascular resistance (Liang et al., 2020), which may *per se* contribute to the occurrence and development of PSVD. In this section, we will review the current literature on the potential interactions between gut microbiome and PSVD, focusing on abnormal intrahepatic porto-sinusoidal vascular microenvironment and hepatic microvascular thrombosis formation.

### 2.1 Gut microbial dysbiosis and porto-sinusoidal microcirculatory dysfunction

The liver contains two distinct microvascular structures. One is made up of continuous endothelial cells organized within the basement membrane, forming a complete vascular structure seen in portal vein blood vessels. The other is composed of discontinuous liver sinusoidal endothelial cells (LSECs) (Xu et al., 2019). The intrahepatic porto-sinusoidal microvascular unit consists of several discrete units, primarily including portal venules, hepatic sinusoids, and central venules. LSECs constitute a natural barrier that separates the liver parenchyma from the bloodstream in the sinusoidal lumen and participate in regulating liver sinusoidal blood flow and material exchange in surrounding tissues, thereby playing a key role in maintaining hepatic microcirculatory homeostasis (Wang et al., 2021c). In addition, LSECs actively regulate intrahepatic coagulation by generating procoagulant factors, stimulating neutrophils, and interacting with platelets (Yang et al., 2017; Hilscher et al., 2019; Gracia-Sancho et al., 2021). The occurrence of PSVD is closely related to changes in the structure and function of the intrahepatic vascular microenvironment (especially liver sinusoids) accompanied by microvascular thrombosis. Portal vein collects blood from the gastrointestinal tract and first supplies it to the capillary network of hepatic sinusoids (Chopyk and Grakoui, 2020). Obstruction of the sinusoids and the resulting increase in hepatic vascular resistance to portal vein blood flow are the main causes of portal hypertension (Mcconnell and Iwakiri, 2018).

Key mechanisms of gut dysbiosis-related alterations in sinusoidal vascular include weakened gut barrier and amplified translocation of PAMPs (Seki and Brenner, 2008). LSECs express a series of scavenger receptors and toll like receptors (TLRs), which render LSECs able to mediate hepatic clearance process of PAMPs and products derived from the gastrointestinal tract (Shetty et al., 2018; Øie et al., 2020). It is well understood that LSECs are exposed to relatively high concentrations of gut-derived PAMPs in portal blood, which can activate TRLs signaling in LSECs further driving chemokine dependent changes and enhancing vasoconstrictor production, increasing portal perfusion pressure (Hilscher et al., 2019). Thus, the phenotype of LSECs exerts pivotal roles in physiological immune functions and maintains liver vascular homeostasis, including regulating porto-sinusoidal shear stress, angiogenesis, as well as hepatic sinusoidal remodeling (Marrone et al., 2016; Gola et al., 2021). LSECs typically exhibit unique phenotypic characteristics, including open fenestrae and lack of a basement membrane. Abnormalities in LSECs distort the normal architecture of the liver and play a key role in the recruitment and activation of platelets, which can lead to microthrombosis and fibrin deposition within the sinusoids (Lisman and Luyendyk, 2018; Abdelmoneim et al., 2010). LSECs capillarization is regarded as an early hallmark in the pathogenesis of portal hypertension (Iwakiri and Groszmann, 2007; Sutton et al., 2018). Meanwhile, the dysfunction of LSECs and the onset of local inflammation which causes damage to small portal vein branches, endothelial dysfunction, activation of HSCs, and hepatic micro-thrombosis, suggest central roles of LSECs in the pathophysiology and onset of PSVD (Zhang et al., 2020; Khanna and Sarin, 2019; Cerda Reyes et al., 2021). Studies have found that increased levels of gut-derived endotoxins and pro-inflammatory cytokines lead to LSECs dysfunction and vasoconstriction via activation endothelin-1 (ET-1), which plays vital roles in raising hepatic vascular resistance (Yadav et al., 2019; Gracia-Sancho et al., 2007). Bacterial infections have also been found to target LSECs, leading to a shift from their normal tolerogenic state to a pro-inflammatory state (Martin-Armas et al., 2006). PAMPS can stimulate the dedifferentiation of LSECs, driving their dysfunction and capillarization (Wilkinson et al., 2020). Leong et al. found that the loss of fenestrations in LSECs was observed in response to bacilli, specifically *Bartonella* bacilli (Leong et al., 1992). Furthermore, liver endothelial cell fenestrations were found to be negatively correlated with a higher abundance of Firmicutes phylum and reduced abundance of Bacteroidetes (Cogger et al., 2016). Taken together, gut microbiota-derived signals and metabolites can influence angiogenesis, transcriptional and metabolic landscape of the hepatic endothelium, thereby shaping the LSECs phenotype (Formes et al., 2021).

In addition to LSECs, other non-parenchymal cells, including hepatic stellate cells (HSCs), monocytes, and Kupffer cells (KCs), are essential for maintaining the function of the liver vascular microenvironment (Cheng et al., 2021). HSCs are wrapped around LSECs and regulate microcirculation within the hepatic sinus through the contractile function of their slender protrusions, thereby affecting the sinusoidal tone and regulating liver blood flow. Activated HSCs increase large amounts of collagen and myofibroblasts which are deposited in the Disse and sinusoidal spaces, exacerbating vascular resistance, which can lead to the distortion of liver vascular structure (Serrano et al., 2019). Additionally, activated HSCs can produce vasoactive mediators such as angiopoietin, vascular endothelial growth factor (VEGF) and increase ET-1 synthesis thereby maintaining the LSEC phenotype or regulating fenestrations, which in turn influences vascular tone and endothelial function, further contributing to hepatic vascular remodeling (Gana et al., 2016; Marrone et al., 2016). In turn, LSECs play an important role in maintaining the quiescence of HSCs and LSECs capillarization can lead to the secretion of exosomes rich in sphingosine kinase-1, promoting the activation of HSCs (Xie et al., 2012). Previous studies have revealed that the LSECs communication with HSCs facilitates sinusoidal vascular remodeling, which is an early feature of intrahepatic portal hypertension (Deleve et al., 2008; Gracia-Sancho et al., 2019, 2008). Intestinal microbiota translocation can cause the activation of HSCs and alter the cell phenotype of LSECs (Cheng et al., 2021; Corbitt et al., 2013; Stojic et al., 2023).

KCs are resident macrophages in the liver sinusoids, playing crucial roles in capturing and eliminating soluble antigens derived from gut microbes via the portal vein. In general, KCs are responsible for sensing and processing gut-derived signals reaching liver sinusoids, such as pathogens, cell fragments, and endogenous metabolites (Zhou et al., 2022b), playing essential roles in pathogen clearance and immunosuppressive features (Li et al., 2022). The expression of TLR receptors on KCs responds to gut-derived LPS exposure, ultimately promoting the release of pro-inflammatory cytokines such as IL-1, IL-6, IL-12, and TNF-α (Mehta et al., 2014; Płóciennikowska et al., 2015). Braedon et al. implicated the gut microbiota as a direct regulator of KCs antibacterial functions (Mcdonald et al., 2020). Moreover, KCs have abilities to enhance LSECs capillarization, resulting in a transition of LSECs morphology toward a more vascular or capillary-like state by losing fenestrations, and forming a distinctive basement membrane (Ford et al., 2015). In cirrhotic patients, KCs activation has been shown to be closely associated with the hepatic venous pressure gradient, liver disease severity, and an increased risk of venous thrombosis (Waidmann et al., 2013; Tranah et al., 2021). CD163 is a monocyte/macrophage specific membrane marker cleaved from the surface of activated macrophages as a soluble form (sCD163) (Maroto-García et al., 2023). KCs serve as the primary source of CD163 in the liver. The activation of KCs during liver fibrosis and inflammation upregulates CD163 expression and promotes the release of sCD163. Consequently, sCD163 levels demonstrate significant potential as a robust biomarker for evaluating the progression of liver fibrosis and the severity of hepatic tissue inflammation (Dultz et al., 2015). Intriguingly, activation of KCs is also observed in PSVD patients, with higher levels of KC activation markers, including soluble CD163 and the mannose receptor, compared to cirrhotic patients (Ørntoft et al., 2021).

In summary, LSECs capillarization, activation of HSCs and KCs as well as hepatic microvascular thrombosis is associated with the dysregulation of vascular homeostasis and increased intrahepatic vascular interactions, which may partly contribute to PSVD progression. Interlinks between different cell types in the porto-sinusoids involve host-produced inflammatory cytokines alongside microbial byproducts generated by the gut microbiota, which affect the hemodynamics of the intrahepatic vascular microenvironment. A deeper understanding of the crosstalk between gut dysbiosis and vascular processes has led to improved insights of the potential microbial mechanisms associated with PSVD.

### 2.2 Gut microbial dysbiosis and hepatic microvascular thrombosis

Coagulation disequilibrium, especially hypercoagulable states or prothrombotic conditions, has been implicated in the PSVD onset and progression (Riggio et al., 2016). Liver biopsy often reveals signs of thrombosis, including intrahepatic portal vein thickening, occlusion, and obstruction in PSVD cases (De Gottardi et al., 2022). Accumulating studies have suggested that the presence of hepatic vein thrombosis may be a common consequence of PSVD, which can be attributed to both reduced portal flow velocity and the elevated prevalence of prothrombotic risk factors (Gioia et al., 2018, 2019). Microvascular thrombosis and platelet aggregation occurring in intrahepatic portal venules and sinusoids are suggested to contribute to PSVD. The gut microbiota can regulate coagulation disorders in thromboembolism (Hasan et al., 2020). Gut dysbiosis characterized by an increased relative abundance of opportunistic pathogenic proteobacteria and fewer beneficial genera play vital roles in thrombosis-related diseases (Xiang et al., 2020; Yin et al., 2015; Yang et al., 2022). Gut microbiota also affects the hemostatic properties of hepatic microvascular endothelium through the gut–liver axis (Kiouptsi et al., 2023). An increasing number of studies have found that gut microbes, PAMPs, and microbial metabolites play important roles in shaping vascular development, affecting endothelial cell function and coagulation system activation causing thrombosis (Hasan et al., 2020; Mohammed et al., 2020). Under gut dysbiosis, gut microbe-derived components into portal-systemic circulation activating PAMPs-induced inflammatory pathways, which are related to prothrombotic states. Gut microbiota-triggered TLR-2 alters the synthesis of von Willebrand factor (vWF) by the liver endothelium and favors platelet integrin-dependent thrombus growth (Jäckel et al., 2017).

LPS, found in the outer membrane of gram-negative bacteria, can influence coagulation and lead to continuous, chronic low-grade inflammation in the liver through stimulation of pattern recognition receptors and TLRs on endothelial cells and platelets, which can culminate in the production of large amounts of inflammatory cytokines and activate the coagulation cascade (An et al., 2022; Vijay, 2018; Ozinsky et al., 2000). LPS has also been reported to prime platelets to respond to activation by common agonists, promoting the expression of tissue factor and exerting prothrombin activity (Reinhardt et al., 2012). *In vitro*, human endothelial cells were incubated were incubated with LPS concentrations similar with those found in the peripheral circulation of liver cirrhosis, and the results showed that LPS increased the release of vWF and factor VIII (Carnevale et al., 2017). Meanwhile, LPS can decrease thrombomodulin expression in LESCs resulting in sinusoidal microthrombus formation and liver dysfunction (Kume et al., 2003). Clinical studies have also found that the imbalance of inflammatory states and gram-negative bacteria-derived products (LPS or other bacterial toxins) leads to activation of the coagulation in cirrhotic patients with PVT (Huang et al., 2023; Georgescu et al., 2023). Mechanistically, translocated LPS derived from the gut microbiota activate the immune response in the liver, triggering inflammatory reactions in the liver, thereby affecting the health of the portal vein and sinusoidal vessels (Violi et al., 2023). *E. coli*-derived LPS has been reported to increase liver damage by inducing macrophage and platelet activation through TLR4 pathway (Carpino et al., 2020). TLR4 is widely expressed in hepatocytes, HSCs, and KCs. Activation of the LPS-TLR4 pathway can potentially become a risk factor leading to liver diseases (Violi et al., 2023).

Previous studies have reported that intestinal translocation of *E. coli* might cause recurrent septic embolization resulting in histological changes similar with those seen PSVD including endothelial damage and obstruction of small portal veins (Kono et al., 1988; Sarin and Aggarwal, 1998; Giuli et al., 2023). Chronic or recurrent infections that cause intestinal antigenemia may ultimately lead to mild portal vein inflammation, resulting in pathological changes compatible with PSVD (De Gottardi et al., 2022; Harmanci and Bayraktar, 2007). In patients with coeliac disease, factors of enteric origin contribute to the obliteration of the portal venous microcirculation, suggesting that prothrombotic factors of gut origin may cause PSVD (Eapen et al., 2011). Gioia et al. found that LPS translocation and the number of TLR4+ macrophages were significantly increased in liver biopsies of patients with PSVD compared to healthy controls. Meanwhile, TLR4+ macrophages were located both in the portal tract and perisinusoidal area, regions typically altered in PSVD with the activation of LPS-TLR4 pathway in patients affected by PSVD (Gioia et al., 2023). The LPS-TLR4 pathway may also be considered a key promoter in the development of PSVD.

## 3 Future research direction and perspectives

**Figure 1:**
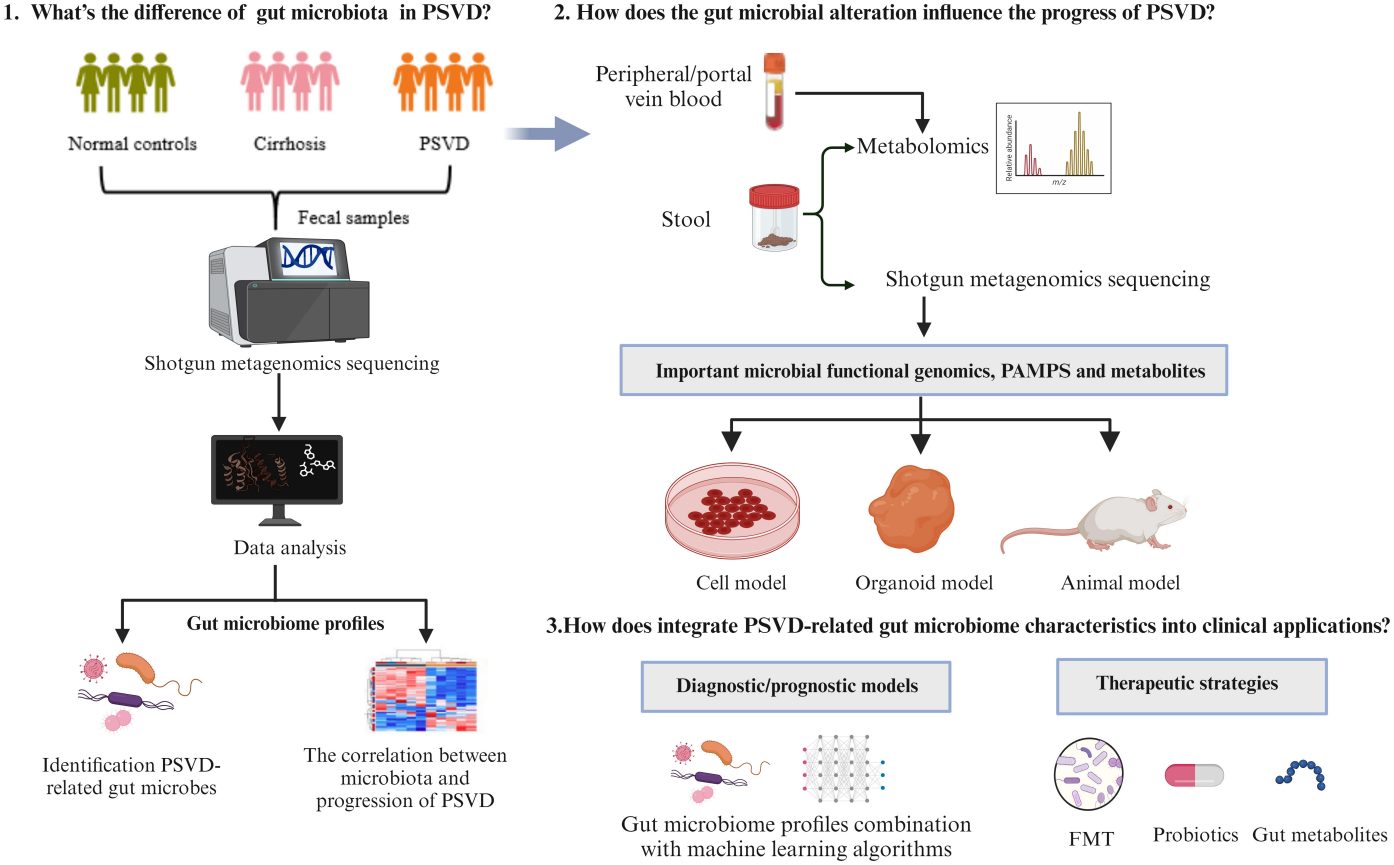
A framework to study interactions between gut microbiome and PSVD.

The changes in gut microbiota and its derivatives on liver pathophysiology has become widely recognized (Wang et al., 2021b; Shen et al., 2023). As the liver is directly supplied by gut-derived blood via the portal vein, the periportal areas would be the first to be exposed to gut-derived metabolites or inflammation substances (English et al., 2021), playing an important role in the function of microvasculature. Based on the extensive evidence linking gut dysbiosis with porto-sinusoidal microcirculatory abnormalities and hepatic thrombosis, it is reasonable to hypothesize that the continuous interaction between gut-derived pathogens and metabolites contributes to the pathophysiology of PSVD. However, the studies on the interactions between gut microbiome and PSVD are limited, and many questions remain unresolved (Figure 1).

A key point is to identify a core group of gut microbes associated with PSVD and to explore how gut dysbiosis impacts the structural and functional changes in in the microbiome that contribute to this condition. The most widely utilized method for classifying and phylogenetically identifying of bacterial community composition is 16S rRNA gene amplicon analysis, which allows for the differentiation of bacteria at the genus level (Rutanga et al., 2018). However, merely understanding the genus and its relative abundance is insufficient for clinical applications or mechanistic research in liver diseases, as each genus encompasses various strains that may exert different pathological or beneficial effects (Giuffrè et al., 2020). As sequencing costs continue to decrease, shotgun metagenomics is progressively replacing 16S rRNA sequencing in microbiome studies. Shotgun metagenomics can identify the composition and structure of gut microbiota—including viruses, bacteria, fungi, and parasites—at the species level, as well as provide insights into microbial gene expression, elucidating the functions of actively expressed genes (Valles-Colomer et al., 2023; Shakya et al., 2019). Although collecting PSVD cases in clinical practice is challenging due to the relatively low prevalence of PSVD and the complex diagnostic process, sufficient sample sizes are essential to account for the inherent inter-individual variability when using shotgun metagenomics to detect alterations in structure and composition of gut microbiota in PSVD patients. Research designs should ensure that the included PSVD patients should be diagnosed based on pathology and clinical manifestations, and that study controls consist of healthy individuals and those with other liver diseases that may be easily confused with PSVD.

Besides unraveling the alterations and interlinks of gut microbiome and PSVD, the pathogenesis of PSVD from the perspective of microbial functional genomics, PAMPs and metabolites is also a key focus requiring further research. Compared to gut microbiota, PAMPs, and microbial metabolites are more readily transported to the liver via the portal vein, where they impact liver function and contribute to liver disease. Metabolomics, which targets metabolites, provides insights into overall metabolic states and host-microbe interactions. A combination of shotgun metagenomics and metabolomics can elucidate the intricate interactions among the gut microbiome, microbial metabolites, and liver diseases. When designing studies on the relationship between gut microbiota, metabolites, and PSVD, it is vital to distinguish between metabolites produced by the host and those generated by microbial communities. Research should focus on identifying microbial metabolites closely linked to PSVD progression, emphasizing the sources of gut microbial metabolites and analyzing the interactions between these metabolites, alterations in microbial communities, and the severity of PSVD. Combining cell models and organoid models with the multi-omics techniques will facilitate functional research and mechanistic exploration targeting PSVD-related pathogenic microorganisms, PAMPs, and metabolites. As illustrated in this review, bacterial LPS accumulation in the liver can induce aberrant characteristics and functions of hepatic sinusoids, promote platelet-dependent hepatic thrombosis, and trigger inflammation, thereby contributing to the onset and progression of PSVD. In addition to LPS, several metabolites have been reported to influence vascular development, affect endothelial cell function, and activate coagulation systems, warranting further investigation, such as trimethylamine oxide (TMAO), short-chain fatty acids (SCFAs), and gaseous molecules (Mohammed et al., 2020; Mitten and Baffy, 2022).

TMAO, a key gut microbiota-derived metabolite, is also associated with inflammation, vascular endothelial injury, and thrombosis (Koeth et al., 2013). Several gut microbes (such as *Desulfovibrio*) can degrade choline, betaine, and L-carnitine from the diet into TMA which is absorbed, transferred to the liver and eventually converted by hepatic flavin monooxygenases into TMAO (Qiu et al., 2018). TMAO acts on inositol-1,4,5-trisphosphate generation in platelets can activate macrophage scavenger receptor expression through various pathways to activate the inflammatory signal pathway, resulting in the aggravation of oxidative stress, endothelial dysfunction, and thrombotic process (Wang et al., 2021a). Studies have revealed that microbial taxa associated with a high choline diet significantly increased TMAO which was positively correlated with enhanced platelet hyper-responsiveness and thrombosis risk (Mohammed et al., 2020; Skye et al., 2018). Higher levels of TMAO can increase endothelial reactive oxygen species (ROS) production and impair vascular endothelial function, which have been found to be positively correlated with thrombosis (Lässiger-Herfurth et al., 2019). Modulating the gut microbiome to target TMAO levels may represent an innovative approach for reducing the risk of thrombosis (Vinchi, 2019). The liver is the main organ responsible for TMAO production, and long-term exposure to high doses may induce chronic liver diseases by modulating inflammatory responses. Indeed, TMAO generated by the gut microbiome affects bile acid metabolism, cholesterol and sterol metabolism, and oxidative stress, promoting the development of metabolic dysfunction-associated fatty liver disease (MAFLD) (Li et al., 2021; Tan et al., 2019). Zhou et al. found that TMAO mediates the crosstalk between the gut microbiota and hepatic vascular niche to affect LSECs characteristics in non-alcoholic steatohepatitis (Zhou et al., 2022a). Nevertheless, there is currently no research available on the relationships between TMAO and hepatic microvascular thrombosis.

Short-chain fatty acids (SCFAs), such as acetate, propionate, and butyrate, which produced through the fermentation of carbohydrate by gut bacteria, are important for maintaining intestinal motility, enterocyte viability, and tight junction integrity (Morrison and Preston, 2016; Boursier et al., 2016). SCFAs in the portal blood participate in the modulation of liver hemodynamics, and the level of circulating SCFAs is negatively related to the severity of liver disease (Koh et al., 2016). Butyric acid has been reported to be reported to inversely associated with the hepatic venous pressure gradient values, and induce inflammatory markers (TNFα and IL-6) in the hepatic, portal, and peripheral blood (Mitten and Baffy, 2022). Inflammation affecting the blood vessels activates the coagulation cascade, promoting the formation of thrombosis (Jonsson and Bäckhed, 2017).

**Figure 2:**
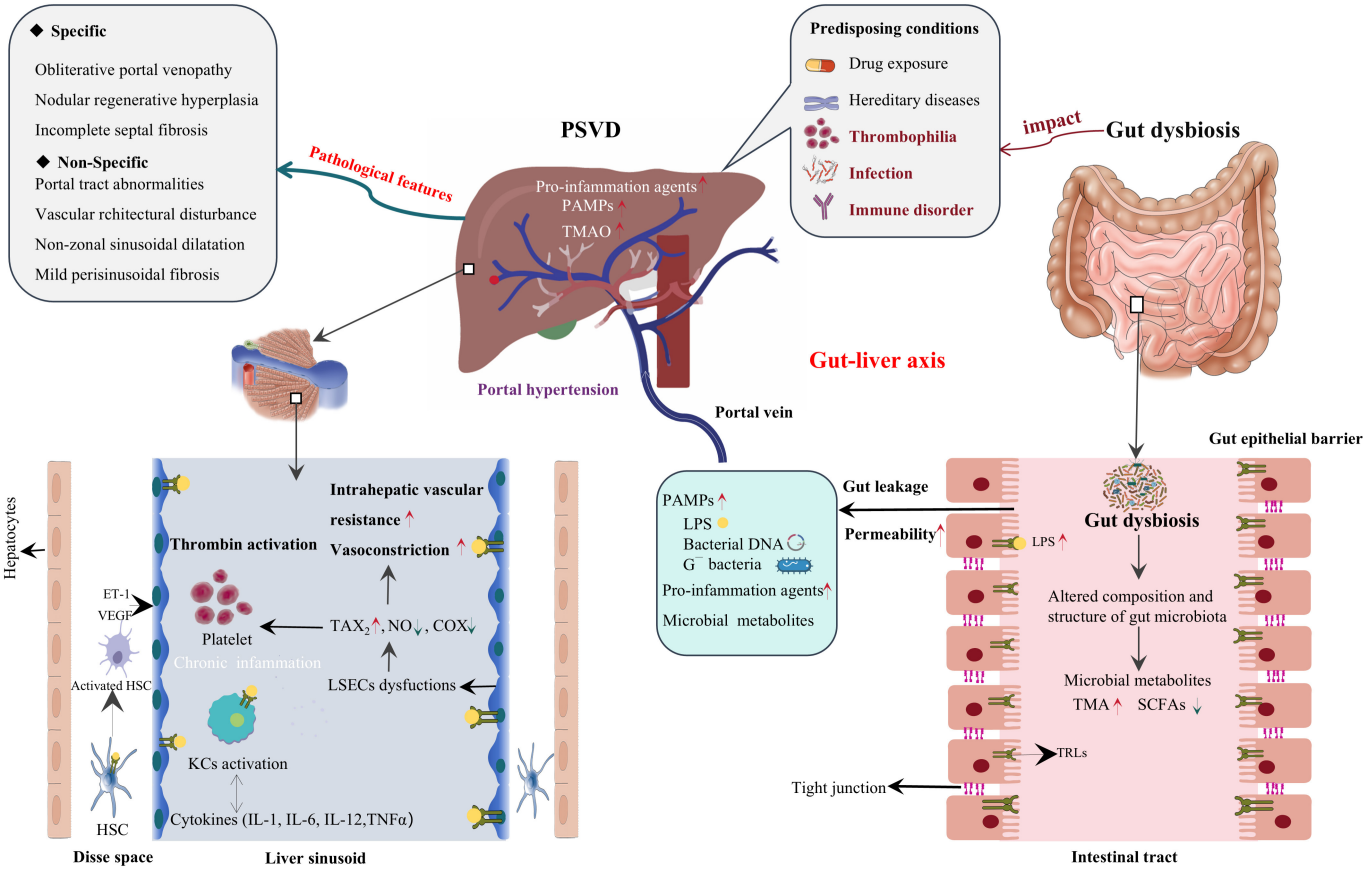
The potential role of gut dysbiosis in the pathobiology of PSVD. Emerging evidence has demonstrated that gut dysbiosis is significantly associated with multiple predisposing conditions of PSVD, particularly immune disorders, bacterial infections and thrombophilia. Changes in gut microbiota, gut-derived bacterial products (PAMPs) and metabolites can stimulate the toll-like receptor (TLRs) signaling, leading to the activate hepatic stellate cells (HSCs), and Kupffer cells (KCs) as well as liver sinusoidal endothelial cells (LSECs) capillarization. These pathophysiological processes trigger the release of pro-inflammatory cytokines and vasoactive substances, which subsequently induce vasoconstriction, elevate intrahepatic vascular resistance, and promote hepatic microvascular thrombosis, ultimately contributing to the development of portal hypertension and other pathological manifestations in PSVD.

Notably, gut microbiota utilizes carbohydrate and protein fermentation, as well as hydrocarbons to produce some gas signal molecules regulation vascular function and maintenance vasculature homeostasis, which is gradually being recognized in liver diseases. Nitric oxide (NO) and hydrogen sulfide (H_2_S) are the best-known gas molecules playing crucial roles in vascular signaling and other processes (Zhou et al., 2022a; Yang et al., 2023). Under normal physiological conditions, endothelial nitric oxide synthase derived NO in the blood can serve as an early marker of endothelial injury (Stanhewicz and Kenney, 2017). LSECs are specialized vascular cells located between the sinusoidal lumen and Disse space. NO is a key factor that maintains the phenotypes of LSECs. Reduced NO bioavailability is associated with liver disease progression, which induces hepatic vascular resistance, activation of HSCs, and endothelial dysfunction in LSECs (Hwang et al., 2023; Poisson et al., 2017). Simultaneously, the ability of abnormal LSECs to synthesize NO is reduced, leading to vasoconstriction, increasing intrahepatic vascular resistance, eventually inducing the development of porto-sinusoidal hypertension (Pillai et al., 2015; García-Pagán et al., 2012). Endogenous H_2_S have demonstrated the involvement in regulating angiogenic responses by activation the VEGF-NO pathway in HSCs participating to maintain LESCs phenotype and functional status (Yang et al., 2023). Together, ecological imbalance in the intestinal flora may promote gut microbes, gut-derived PAMPs, and microbial metabolites to act on porto-sinusoidal vascular abnormalities and hepatic microthrombosis, thus participating in the occurrence and development of PSVD (Figure 2).

Understanding the links between gut microbes and PSVD not only helps us better understand the pathophysiological mechanisms of PSVD, but also provides important information for clinical diagnosis and treatment. Currently, in addition to exploring the diagnostic and prognostic value of microorganisms for diseases, targeting the gut microbiota has become the focus of emerging therapies with varying success such as fecal microbiota transplantation, probiotics, selective antibiotic use, and targeted small metabolites produced by gut microbiota, such as SCFAs and TMAO (Wong and Levy, 2019; Velasquez et al., 2016). Advances in gut microbiota sequencing technology and metabolomics have made the detection of specific changes in gut microbiota and related metabolites valuable methods for non-invasive diagnostic or prognostic biomarkers of PSVD. Previous studies have identified metabolic features that clearly differentiate patients with PSVD from those with cirrhosis and portal hypertension, as well as from healthy individuals (Seijo et al., 2013, 2016). However, specific treatments to prevent disease progression in PSVD patients are currently unavailable. Therapeutic options are limited to agents addressing complications related to portal hypertension and hepatic thrombosis, which generally result in a poor prognosis. With better understanding of the interactions between gut microbial alterations and PSVD, identifying PSVD-related characteristic gut-derived microbes and metabolites may provide promising research fields for related clinical applications.

## 4 Conclusion

Accumulating evidence suggests that gut dysbiosis may play a pivotal role in the occurrence and progression of PSVD by promoting porto-sinusoidal abnormalities and intrahepatic thrombosis. Gut dysbiosis disrupts the homeostasis of the gut-liver axis, facilitating the translocation of gut-derived microbial components, including LPS and metabolites into the liver. These factors induce structural and functional abnormalities in LSECs, HSCs, and KCs, while simultaneously promoting intrahepatic vascular resistance and coagulation dysregulation within the hepatic sinusoids, leading to the subsequent progression of PSVD. Currently, there is still limited understanding of the direct association between gut microbiome and PSVD. Integrating gut microbiota research into the clinical management of PSVD holds significant promise for enhancing diagnostic accuracy, prognostic evaluation, and therapeutic efficacy. Future research should focus on identifying specific gut microbial signatures and metabolites associated with PSVD by utilizing advanced multi-omics approaches. Elucidating the mechanistic pathways through which gut-derived signals, especially microbial functional genomics and metabolites, influence PSVD will be crucial for developing innovative, non-invasive diagnostic tools and personalized treatment strategies for PSVD.
